# CRONE PEDAGOGIES AND THE EMANCIPATORY POTENTIAL OF HISTORICAL MEMORY

**DOI:** 10.1093/geroni/igae098.1740

**Published:** 2024-12-31

**Authors:** Nicholas DiCarlo

**Affiliations:** Hunter College / UCSF Emancipatory Sciences Lab / Mountain School of Psychoanalysis, New York, New York, United States

## Abstract

Crone pedagogies (DiCarlo, 2023) embrace abject identities as epistemoloical standpoints of wisdom. This emerging pedagogical tradition centers mourning loved ones, ancestors, and activist who shaped us and teaches an attentive respect for the ghosts that haunt us and pull at normative unconscious processes (Layton, 2019). Gerontological study provides a unique point of inquiry into how we carry knowledge across the lifespan and through generations, as ageism stereotypes, medicalizes, and segregates older adults– collapsing complex personhoods into tropes and units of business. This presentation features examples from teaching first year MSW students about ageism, decoloniality, and abolitionist movements. Historical memory positions teacher-learners with opportunities to celebrate and mourn the activists and scholars who are shaping the fight against policing, transphobia, and displacement. Crone pedagogies underscore the necropolitical dimensions of grief and grievability (Butler, 2009), and encourage critical reflection on survival in oppressive systems and collective praxes of resistance.

